# Supplementary material for: Mapping the inter- and intra-genic codon-usage landscape in Homo sapiens
Source: NAR Genom Bioinform. 2026 Mar 3;8(1):lqag024. doi: 10.1093/nargab/lqag024 (PMC12954173; doi:10.1093/nargab/lqag024)
Supplement: lqag024_Supplemental_Files [file lqag024_supplemental_files.zip › Full_Figure_S3.pdf]

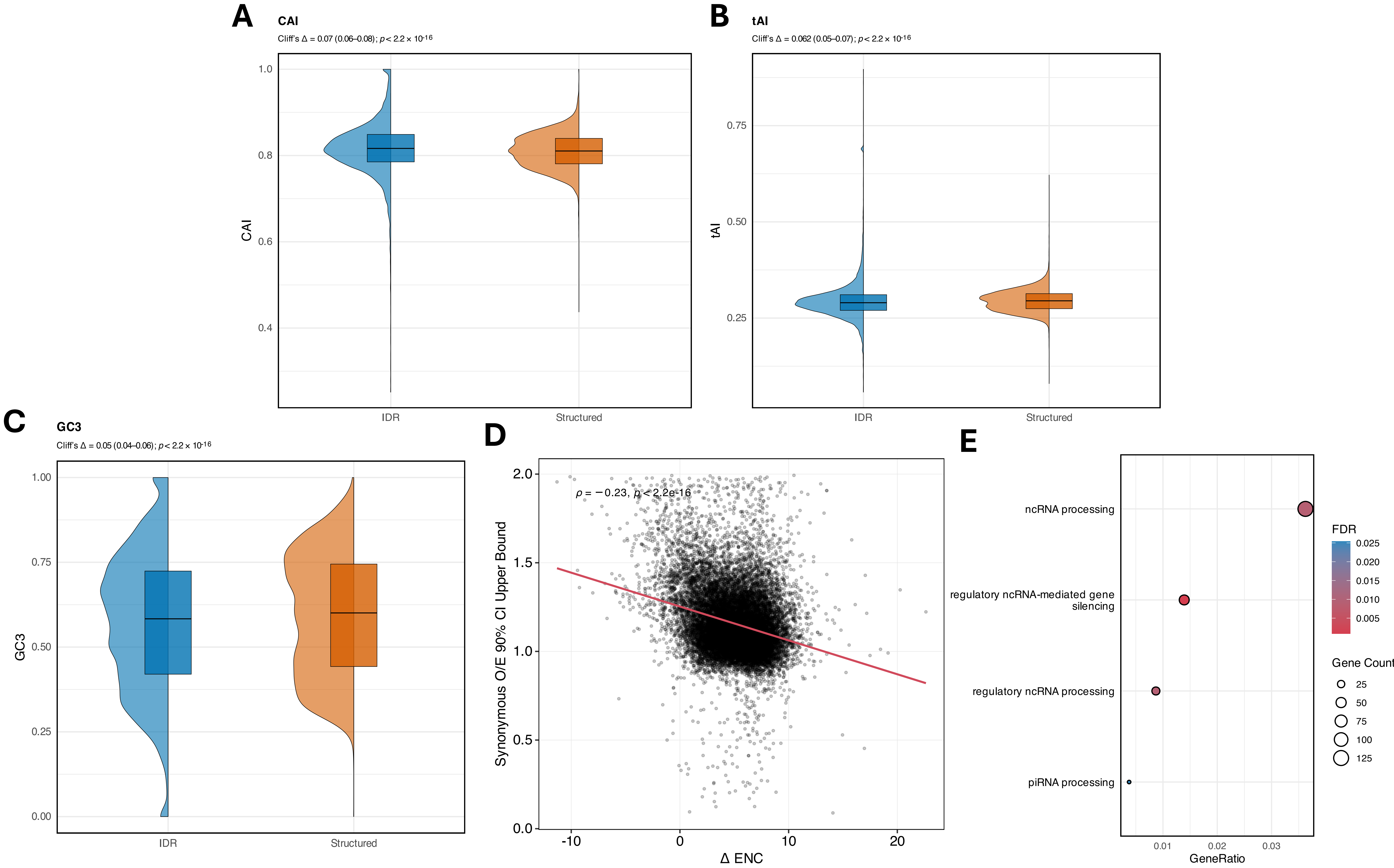

Figure S3. (A) Violin and box plots of CAI in IDRs (blue) versus structured regions (orange). Median CAI values are nearly identical, and although the difference is statistically significant, the effect size is negligible ($p < 2.2 \times 10^{-16}$; Cliff's Δ = 0.07). **(B)** Violin and box plots of tAI show similar distributions in IDRs and structured regions ($p < 2.2 \times 10^{-16}$; Cliff's Δ = 0.06). **(C)** Violin and box plots of GC3 reveal comparable GC3 content between IDRs and structured regions ($p < 2.2 \times 10^{-16}$; Cliff's Δ = 0.05). **(D)** Scatterplot of ΔENC versus the upper bound of the 90% CI for synonymous O/E shows a negative correlation (ρ = –0.23; $p < 2.2 \times 10^{-16}$), indicating that genes with stronger codon bias tend to have lower synonymous constraint scores. (**E)** Top enriched GO biological process terms for unbiased control genes. Dot size represents gene count and color indicates significance (FDR).
