# Supplementary material for: Mapping the inter- and intra-genic codon-usage landscape in Homo sapiens
Source: NAR Genom Bioinform. 2026 Mar 3;8(1):lqag024. doi: 10.1093/nargab/lqag024 (PMC12954173; doi:10.1093/nargab/lqag024)
Supplement: lqag024_Supplemental_Files [file lqag024_supplemental_files.zip › S1_A-J_Full.pdf]

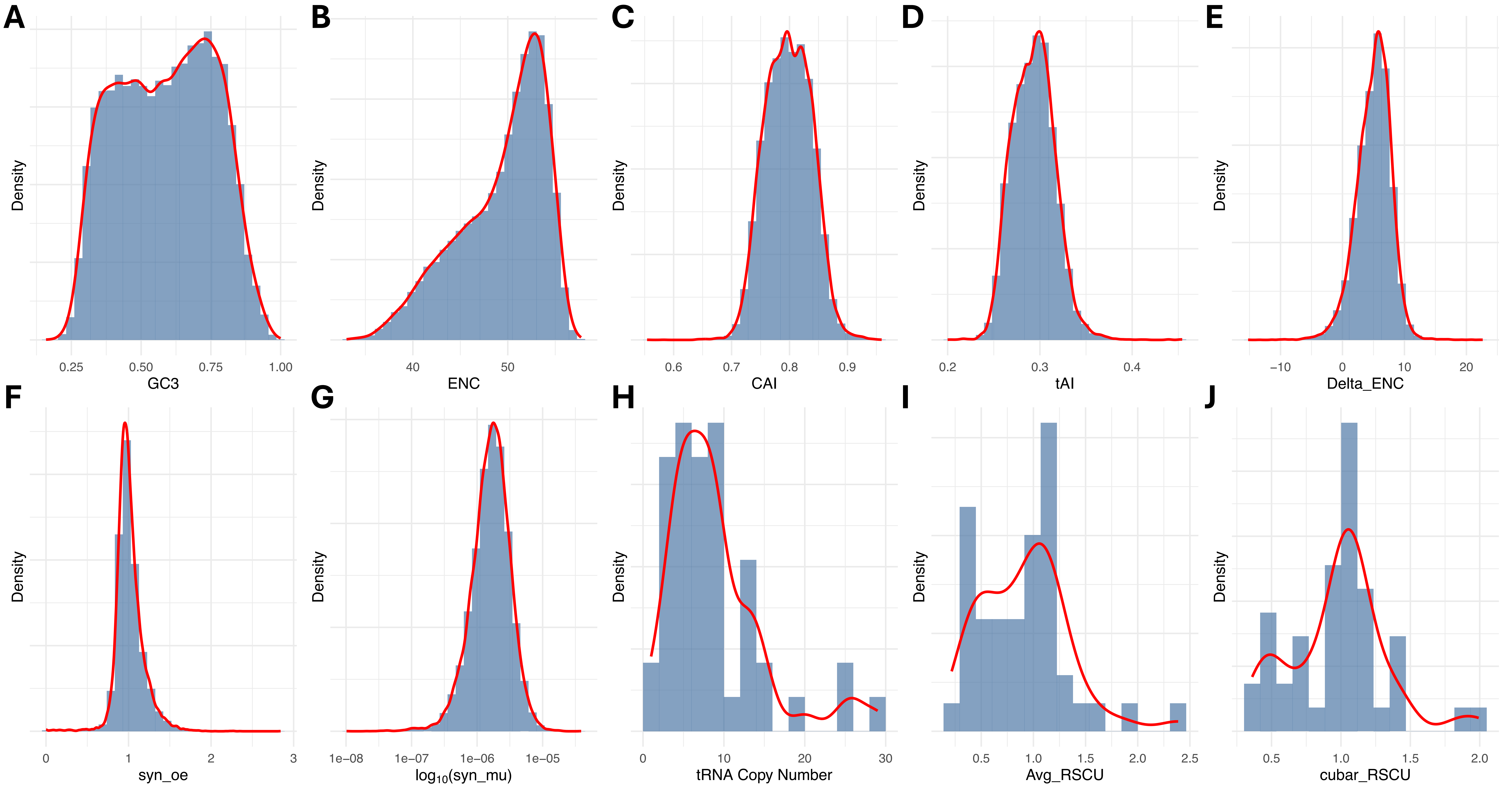

Figure S1. Genome-wide distributions of codon usage metrics. Histograms with overlaid kernel density estimates (red lines) showing the data structure for the variables analyzed in this study. **Top Row: (A)** GC content at the third synonymous position (GC3); **(B)** Effective Number of Codons (ENC); **(C)** Codon Adaptation Index (CAI); **(D)** tRNA Adaptation Index (tAI); **(E)** ΔENC (deviation of ENC from neutral expectation). **Bottom Row: (F)** Synonymous observed/expected ratio (syn_oe), acting as a proxy for selective constraint; **(G)** Synonymous mutation rate log10 scale; **(H)** tRNA gene copy number; **(I)** Average Relative Synonymous Codon Usage (RSCU) calculated per gene/outlier genes removed; **(J)** CUBAR RSCU calculated genome-wide using CUBAR.
