# Supplementary material for: Mapping the inter- and intra-genic codon-usage landscape in Homo sapiens
Source: NAR Genom Bioinform. 2026 Mar 3;8(1):lqag024. doi: 10.1093/nargab/lqag024 (PMC12954173; doi:10.1093/nargab/lqag024)
Supplement: lqag024_Supplemental_Files [file lqag024_supplemental_files.zip › S2_A-J_Full.pdf]

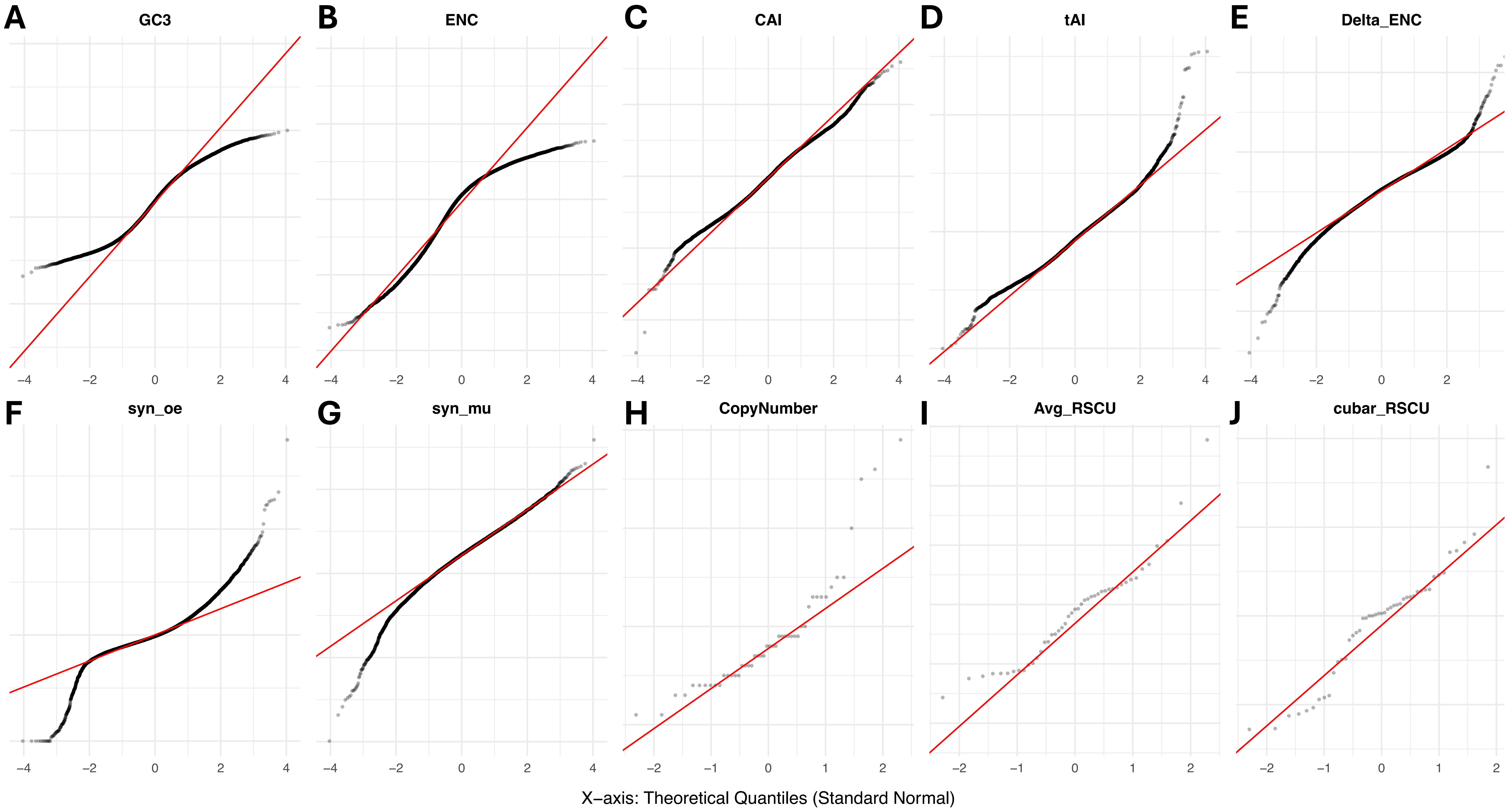

Figure S2. Quantile-Quantile (Q-Q) plots assessing the normality of genome-wide variables. Each panel displays a Q-Q plot comparing the distribution of the observed data against a theoretical standard normal distribution. The X-axis represents the theoretical quantiles, and the Y-axis represents the observed sample quantiles for each metric. The red solid line serves as a reference for perfect normality; points falling along this line indicate a normal distribution. **Top Row: (A)** GC content at the third synonymous position (GC3); **(B)** Effective Number of Codons (ENC); **(C)** Codon Adaptation Index (CAI); **(D)** tRNA Adaptation Index (tAI); **(E)** ΔENC (deviation of ENC from neutral expectation). **Bottom Row: (F)** Synonymous observed/expected ratio (syn_oe), acting as a proxy for selective constraint; **(G)** Synonymous mutation rate log10 scale; **(H)** tRNA gene copy number; **(I)** Average Relative Synonymous Codon Usage (RSCU) calculated per gene/outlier genes removed; **(J)** CUBAR RSCU calculated genome-wide using CUBAR.
